# Molecular Diagnostics for Group A Streptococcal Pharyngitis: Clinical and Economic Benefits in the Belgian Healthcare Context

**DOI:** 10.3390/jcm13216627

**Published:** 2024-11-04

**Authors:** Mohammad Hossein Panahandeh, Reza Soleimani, Yasmine Nezzar, Hector Rodriguez-Villalobos, Benoît Kabamba-Mukadi, Alexandre Grimmelprez, Patricia Schatt

**Affiliations:** 1 Microbiology Laboratory, Department of Laboratory Medicine, Clinique Notre-Dame De Grâce, 6041 Gosselies, Belgium; 2 Microbiology Laboratory, Department of Laboratory Medicine, Centre Hospitalier Universitaire Ambroise Paré (HELORA—Site Kennedy), 7000 Mons, Belgium; 3 Microbiology Laboratory, Departement of Laboratory Medicine, Cliniques Universitaires Saint-Luc, 1200 Brussels, Belgium

**Keywords:** NAAT, RADT, *Streptococcus* A, pharyngitis, ID NOW, OSOM

## Abstract

(1) Background: Group A Streptococcal (GAS) pharyngitis is common, resulting in numerous ambulatory visits. Accurate diagnosis is challenging. This study evaluated the clinical utility, cost, and performance of a nucleic acid amplification test (NAAT) for GAS detection, comparing it to a rapid antigen detection test (RADT) and throat culture. Additionally, we assessed the diagnostic stewardship related to these testing methods to ensure appropriate antibiotic use in clinical practice. Methods: Between November 2022 and February 2023, 82 throat swabs were analyzed, with McIsaac clinical scores calculated for each. The Abbott ID NOW STREP A 2 NAAT and Sekisui Diagnostics’ OSOM^®^ STREP A RADT were performed, followed by bacterial culture. Diagnostic performance was compared using culture as the gold standard. Results: Of the 82 samples, 28 (34.14%) tested positive for pathogenic germs, primarily *Streptococcus* pyogenes (92.85%). RADTs showed a sensitivity of 80.76% and a specificity of 100%, while NAATs demonstrated a sensitivity of 100% and specificity of 96.42%. Cost analysis indicated the need for reimbursement adjustments to optimize NAAT’s economic benefits. Clinical data indicated that symptoms alone were insufficient for reliable diagnosis. Conclusions: This study confirmed the superior sensitivity of Abbott’s Strep A2 NAAT over RADT. Given the Belgian guidelines against routine antibiotic treatment for pharyngitis and considering local treatment recommendations and cost, implementing NAAT for GAS detection in Belgian laboratories is less beneficial. However, the role of NAAT in supporting antimicrobial stewardship by ensuring appropriate antibiotic use remains significant.

## 1. Introduction

Pharyngitis accounts for 1 to 2 per cent of all ambulatory care visits [1]. School-aged children experience the highest incidence rate [2]. While viruses are the most common cause of pharyngitis, approximately 5% to 15% of adult cases are caused by Group A *Streptococcus* (GAS) [3]. Notably, during a one-year sample collection, 18.96 percent of throat swabs sent to our laboratory tested positive for GAS. Unfortunately, there is no single sign or symptom with a high-enough probability ratio to diagnose GAS accurately. The modified Centor criteria, which rely on age and up to four clinical features (fever, tonsillar exudates, swollen tender anterior cervical nodes, and the absence of cough), are among the most frequently used prediction rules in adults and children. Nevertheless, even when all four criteria are present, the probability of GAS infection is about 55.7% [4]. Based on local/national recommendations, each country adopts a different strategy for managing pharyngitis. If the chosen strategy involves antibiotic treatment, it is generally advised to confirm the presence of GAS through laboratory testing [5].

The definitive method for diagnosing GAS pharyngitis in a laboratory setting is through the bacterial culture of a throat swab, considered the gold standard. However, the practicality of this approach is limited due to the significant delay between specimen collection and the final microbiological diagnosis, which could hamper effective management [6].

Rapid antigen diagnostic tests (RADTs) are a potentially more feasible alternative because of their quick turnaround time and the ability to avoid unnecessary antibiotic prescriptions [6]. Due to their relatively lower sensitivity compared to throat culture, the American guidelines suggest that a throat culture should confirm negative RADT results in children and adolescents to minimize the possibility of missing positive cases of GAS pharyngitis [4,7]. In adults, a backup culture is typically not required due to the generally lower incidence of GAS pharyngitis and the lower risk of developing rheumatic fever [8]. Molecular assays in detecting GAS pharyngitis are as sensitive as either culture alone or RADTs with reflexive culture and, therefore, do not need a follow-up throat culture [9,10].

Data from 2012 to 2022 from the National Reference Center for GAS in Belgium show that GAS resistance to erythromycin and clindamycin has remained stable over the years, staying below 10%. Resistance to tetracycline, despite an increase during the COVID period, returned to pre-COVID levels of around 12% in 2022. To date, no penicillin-resistant strains have been reported [11].

According to Belgian recommendations, it is generally not necessary to administer antibiotics for pharyngitis (even if it is caused by GAS), so identifying the responsible germ does not provide added value as a first-line approach [12,13,14]. However, our experience shows that many general practitioners request confirmation of the absence of GAS. Primary care samples constitute approximately 85% of our laboratory’s total activity. When physicians send a throat swab, we perform either a culture alone or a culture and RADT, based on their prescription. Patients and clinicians increasingly recognize and appreciate rapid results, especially after the COVID-19 pandemic.

NAATs are known for their high sensitivity (unlike RADTs) and fast results (unlike culture). In this study, we aimed to evaluate the added value of incorporating ID NOW™ STREP A 2 (Abbott, IL, USA), a molecular isothermal test based on NEAR (nicking enzyme isothermal amplification reaction) that detects the *cepA* gene encoding the C5-peptidase in GAS. We considered its benefits, costs, and performance compared to RADT and throat culture while also understanding the sample management strategies of physicians collaborating with our laboratory in a Belgian healthcare setting.

## 2. Materials and Methods

Conducted at the hospital Notre-Dame de Grâce in Gosselies, which is located in the province of Hainaut, the study analyzed 82 throat swab specimens, each containing 1 mL of liquid Amies transport medium (ESwab^®^ Beckton Dickinson, NJ, USA), collected between November 2022 and February 2023 (female: 52/82). These samples were accompanied by a clinical information form completed by clinicians who were informed about the study and agreed to participate during the collection period. Laboratory personnel in the microbiology service analyzed the samples by conducting three tests, all performed using the same liquid Amies from each swab. The transport medium was more than sufficient for all three tests, with no interference between them, as sterile swabs were used for each test.

The swabs were briefly (about 10 s) vortexed, and then a molecular assay using Abbott’s ID NOW STREP A 2 was performed, following the instructions provided in the manufacturer’s package insert [10]. The RADT was conducted using the OSOM^®^ STREP A (Sekisui Diagnostics, MA, USA), according to the manufacturer’s guidelines [15]. For the culture, a BD BBL™ Columbia Agar with 5% Sheep Blood (Becton Dickinson, NJ, USA) was inoculated using the semi-quantitative quadrant streaking method and incubated at 37 °C under CO_2_. In this technique, the inoculum is spread sequentially across four quadrants to progressively dilute the bacterial concentration. The semi-quantification of bacterial colonies was determined based on the extent of growth in the quadrants: one cross (1+) was assigned if colonies were present only in the first quadrant, while four crosses (4+) were given if colonies densely populated all four quadrants.

Only the colonies exhibiting a hemolytic effect on the blood and displaying a compatible beta-hemolytic aspect characteristic of *Streptococcus* were selected for identification using matrix-assisted laser desorption/ionization-time of flight mass spectrometry (MALDI-TOF) on MALDI Biotyper^®^ sirius System (Bruker, MA, USA). The results obtained from the RADT and NAAT were compared to the throat culture, which is widely regarded as the reference method for diagnosing bacterial pharyngitis. Additionally, the clinical forms accompanying the samples contained comprehensive data regarding the patients’ symptoms at the time of sampling, along with the physician’s management strategy and the specific type of antibiotic prescribed, if any.

Data regarding throat culture results over an entire year were extracted to be compared with the percentages of the results from our study.

Statistical analyses were carried out using SPSS 29.0.1 software. Descriptive analyses (frequencies, means, and 95% CIs) were performed on the data. The data were compared using the chi-square test for ordinal variables. A *p*-value of less than 0.05 was considered significant.

Our study fulfilled the ethical principles provided by the Declaration of Helsinki and was approved by the Local Medical Ethics Committee (Ref: OM040). All participants gave their informed consent.

## 3. Results

### 3.1. Population

During the sample collection period for the study, a total of 82 samples were analyzed according to the study protocols. The average age of the patients was 15 years (95% confidence interval [CI]: 11.37 to 18.71), with 52 females. The samples originated from 18 different doctors (two pediatricians and 16 general practitioners), mainly from the Charleroi region, who routinely requested rapid tests for suspected bacterial pharyngitis.

### 3.2. Culture Results

Of the 82 samples analyzed, 28 (34.14%) were positive for bacteria relevant to throat infections. Specifically, 26 samples (92.85%) were positive for Group A *Streptococcus* (GAS) and 2 samples (7.14%) for Group G *Streptococcus* (GGS). These results are consistent with data obtained in our laboratory over one year (from March 2022 to February 2023), where 726 out of 3316 throat swab cultures (21.89%) were positive. Of these, 629 cultures (86.63%) were positive for GAS and 97 cultures (13.36%) for GGS (data non-published).

### 3.3. Test Performance

To compare the performance of the RADT and NAAT against throat culture, only samples in which the cultures were positive for GAS (n = 26/82, 31.70%) were reported as culture positive; the others were considered culture negative. The results are presented in Table 1.

The analytical sensitivity (aSn) of the RADT in detecting GAS was 80.76% (95% CI: 62.12% to 91.49%), and the analytical specificity (aSp) was 100% (95% CI: 93.58% to 100.00%). The positive predictive value (PPV) was 100% (95% CI: 84.54% to 100.00%), and the negative predictive value was 91.07% (95% CI: 82.21% to 96.45%). Higher bacterial loads on the swabs corresponded to an increased likelihood of positive RADT result (*p* < 0.001, Table 2). As mentioned earlier, a negative RADT result could not rule out the presence of GAS. On the other hand, the aSn of the NAAT was 100% (95% CI: 87.13% to 100.00%); however, its aSp was 96.42% (95% CI: 87.88% to 99.02%). The PPV was 92.86% (95% CI: 77.35% to 98.02%), and the NPV was 100% (95% CI: 93d.36% to 100.00%).

Table 2 presents the RADT positivity rates in relation to the semi-quantitative assessment of microbial growth on throat culture media, based on the semi-quadrant streaking method. Statistical analysis revealed a significant association between RADT positivity and the extent of microbial growth on the culture medium (*p* < 0.001). In simpler terms, higher bacterial loads on the swabs correlate with an increased likelihood of a positive RADT result.

### 3.4. Turnaround Time (TAT)

The time required for receiving and dispatching swabs remained consistent across all three methods. Time comparisons are made starting from when laboratory personnel receive the swab and begin sample analysis. RADT typically takes approximately seven minutes, slightly less than the total time required for NAAT, which involves approximately two minutes for sample manipulation/preparation plus six minutes for analysis of negative results or less for positive results. RADT allows for simultaneous analysis of multiple samples. In contrast, the ID NOW NAAT instrument processes samples sequentially, which may limit throughput, particularly when handling multiple samples arriving from primary care practitioners. However, other NAATs exist that offer rapid sample-to-answer testing, such as the Cepheid’s Xpert^®^ Xpress Strep A [16] and DiaSorin’s Simplexa^®^ Group A Strep Direct [17], which do not have this limitation, as they allow for simultaneous analysis of multiple samples.

Although throat swab culture is not the most time-consuming laboratory microbiological process, it is considerably more labor-intensive than rapid tests. Inoculating a 5% sheep blood agar took less than a minute. However, the culture required at least 24 h incubation before results were available. After incubation, an experienced laboratory technician examined the culture media, selected colonies displaying the characteristic beta-hemolytic aspect of *Streptococcus*, and performed identification using MALDI-TOF MS, which could take up to 11 min.

### 3.5. Cost

The parameters considered in the cost calculation are shown in Table 3. It is important to note that costs may be lower than those displayed after negotiations between firms and laboratories. However, generally speaking, the most cost-effective method is culture alone, followed by culture with RADT. In Belgium, methods involving NAAT are not considered cost-effective due to the absence of reimbursement by insurance, in contrast to some other countries where such tests may be covered.

### 3.6. Patients’ Clinical Symptoms and Signs

Based on patients’ symptoms, a McIsaac score was calculated. Table 4 illustrates the correlation between the later score, throat culture (TC) results, and practitioners’ management. A McIsaac score of 2 or higher shows a low PPV of 39.44% (CI 95%: 28.07% to 50.80%) and a high NPV of 90.91% (CI 95%: 73.92% to 107.90%) (compared to 26.34% and 96.31%, respectively, in the McIsaac et al.’s study [18]) for detecting GAS. However, increasing the McIsaac score threshold to 3 raises the PPV slightly to 47.83% (CI 95%: 33.39% to 62.26%), while decreasing the NPV to 80.56% (CI 95%: 67.63% to 93.48%). In other words, clinical symptoms alone cannot reliably distinguish between GAS and viral pharyngitis. A McIsaac score below 2 can help reduce further testing or unnecessary antibiotic prescriptions.

In a total of 51 out of 82 clinical cases, which represents approximately 62.19%, physicians made the decision to opt for a therapeutic approach to treatment. Among the various treatment options available, the most frequently prescribed medications were amoxicillin and azithromycin. Specifically, amoxicillin was utilized in 37 distinct cases, and notably, 67.56% of these instances involved children and adolescents who were under the age of 14 years. Meanwhile, azithromycin was prescribed in 12 separate cases, with 91.66% of these prescriptions also falling within the same age group of children and adolescents under 14 years old.

In addition to these commonly used antibiotics, both amoxicillin–clavulanic acid and cefadroxil were each prescribed once, with both prescriptions targeting children and adolescents who were under 14 years of age. It is important to highlight, as mentioned earlier in our discussion, that since clinical symptoms are not considered highly sensitive in effectively distinguishing between bacterial and non-bacterial origins of infection, only 42.10% of cases and 50.0% of cases, respectively, demonstrated positive culture results for GAS when utilizing empirical treatment and test-based treatment strategies.

## 4. Discussion and Conclusions

Our study confirmed the superiority of Abbott’s Strep A2 assays over RADT in terms of sensitivity, as shown in other similar studies [19,20]. Indeed, we found a sensitivity of 100% (CI 95%: 87.13% to 100.00%) and a specificity of 96.42% (CI 95%: 87.88% to 99.02%) for the Abbott Strep A2 ID NOW, compared to the OSOM^®^ STREP A test, which had a sensitivity of 80.76% (CI 95%: 62.12% to 91.49%) and specificity of 100% (CI 95%: 93.58% to 100.00%) in our study. The sensitivity of RADT depends on the inoculum size, as shown in other studies [21].

Our findings underscore the complexity of diagnosing bacterial pharyngitis, where molecular assays frequently detect a greater number of true positive cases in symptomatic patients [22] as well as cases of asymptomatic carriers [23]. We observed two cases with negative RADT and culture but positive NAAT results, both with a McIsaac score below 2, suggesting a low probability of GAS infection. It should be noted that 93.1% of cultures become negative one day after treatment, whereas 20% of NAATs can remain positive up to two weeks after treatment [24,25]. This underlines the necessity of interpreting NAAT and culture results in the context of clinical symptoms and history to avoid inappropriate treatments.

While NAAT’s high sensitivity may eliminate the need for bacterial culture confirmation, we emphasize that GAS is a significant cause of bacterial pharyngitis but not the only bacterial etiology. Our study found that 7.14% of the positive throat culture isolates were pathogenic bacteria not detected by rapid diagnostic methods, accounting for approximately 13.36% of positive cultures in our laboratory over the past year. Group G *streptococcus*, detected in 2.92% of our throat cultures annually, affects primarily college students and young adults, often linked to community and food-borne outbreaks [26]. Pharyngitis caused by Group G *streptococcus* is clinically indistinguishable from that caused by GAS. These relevant germs among others could be missed if throat cultures are abandoned.

There is notable variability in the diagnostic criteria for GAS among countries, with some employing clinical criteria, scores, or rapid tests exclusively. European and North American recommendations endorse the use of RADT depending on clinical scores, with the exception of Mexico, England, the Netherlands, and Belgium, where the use of RADT is not included in their recommendations [27]. The Belgian Antibiotic Policy Coordination Committee (BAPCOC) [13] and the Dutch Society of General Practitioners (NHG) [28] recommend against using clinical scores and rapid tests to decide on antibiotic prescriptions for acute sore throat. Additionally, they consider it irrelevant to differentiate between bacterial and viral origins [12,13,14,28]. The debate on the necessity of antibiotics for uncomplicated GAS infections continues, with the Number Needed to Treat (NNT) for preventing abscesses exceeding 150 and even higher for otitis media and sinusitis, conditions typically resolving without antibiotics. Antibiotics offer a modest symptom duration reduction of about 16 h on average [29].

The impact of antibiotics on reducing non-suppurative complications from GAS is controversial. While some studies indicate a 70% reduction in non-suppurative complications with antibiotic treatment [30], others attribute this reduction to decreased rheumatogenicity of GAS strains [31]. In Belgium, only 13% of invasive GAS infections originate from the respiratory tract, and the incidence of ARF is low [32].

Our study demonstrates the superior diagnostic performance of Abbott’s Strep A2 NAAT for detecting GAS, yet certain limitations need to be acknowledged. The sample size of 82 specimens may limit the statistical power and generalizability of the findings. Additionally, the lack of comparison with other NAAT platforms restricts a more comprehensive evaluation of their sensitivity and specificity. Further research is needed to assess NAAT performance across a broader range of clinical settings and patient populations, including asymptomatic carriers, to better understand its role in managing GAS infections and contributing to antimicrobial stewardship.

Despite the clear advantages of NAAT in detecting GAS, the widespread routine use of microbiological testing for pharyngitis in Belgium remains contentious. Belgian guidelines, such as those from BAPCOC [13], advise against routine antibiotic use for pharyngitis, especially in uncomplicated cases, where antibiotics offer minimal clinical benefit. Therefore, microbiological testing should not significantly alter clinical management in most patients, given the limited indications for antibiotics. However, despite these recommendations, general practitioners frequently request diagnostic tests to confirm or rule out Group A Streptococcus infections. This reflects a divergence from guidelines, driven by a desire to ensure appropriate treatment and alleviate patient concerns.

This discrepancy arises in part due to physician and patient expectations, particularly in the post-COVID era. The pandemic has heightened the demand for diagnostic certainty, and both patients and healthcare providers have grown accustomed to rapid, definitive testing. Our study reflects this trend, with both throat cultures and rapid tests being routinely performed upon clinician request, despite the limited necessity for antibiotic therapy. Diagnostic stewardship plays a pivotal role in addressing this issue, ensuring that testing is conducted in line with national guidelines to reduce unnecessary diagnostics and prevent the overuse of antibiotics, which remains a key contributor to the global problem of antimicrobial resistance.

It is important to recognize that treatment decisions should not be based solely on the distinction between bacterial and viral pharyngitis. A more nuanced approach, as recommended by Belgian guidelines [13], should focus on identifying high-risk patients. General practitioners must assess individuals with greater susceptibility to complications, such as immunocompromised patients; those with chronic illnesses; and patients with a history of rheumatic fever, recent prosthetic surgery, or heart valve disorders. In these cases, appropriate microbiological testing can guide targeted treatment decisions. Moreover, severely ill patients presenting with significant clinical symptoms should also be considered for further diagnostic evaluation.

In this context, the high sensitivity of NAAT plays a crucial role in improving diagnostic stewardship. By accurately identifying GAS infections, NAAT enables clinicians to make more informed decisions, reducing the overuse of antibiotics for viral or non-bacterial pharyngitis. This targeted approach aligns with the broader goal of combating antimicrobial resistance, a key concern in modern healthcare. Furthermore, NAAT can help avoid unnecessary healthcare costs associated with misdiagnosis or overprescription, while still providing rapid results that benefit both patients and clinicians. By ensuring that antibiotics are reserved for confirmed bacterial infections, NAAT contributes to both improved patient care and more efficient resource use within the Belgian healthcare system.

In our study, 39% of the therapeutic strategies were influenced by microbiological results, indicating that these results continue to play a substantial role in clinical decision-making, particularly with regard to antibiotic prescription. The reliance on microbiological findings for guiding treatment is well-documented, with clinicians frequently using test results to inform their antibiotic prescription practices [33]. Microbiology laboratories, therefore, remain integral not only in diagnosis but also in supporting antimicrobial stewardship efforts. By contributing to surveillance systems that monitor antibiotic efficacy and resistance patterns, laboratories play a key role in informing policy and clinical practice [34].

However, it is essential to emphasize that microbiological testing should be reserved for cases where it is truly warranted. Overuse or inappropriate use of these tests can lead to unnecessary treatments, increased healthcare costs, and potentially misleading results. Thus, adherence to proper guidelines is critical to ensuring that testing is applied judiciously. In cases where testing is necessary, timely communication between the laboratory and the clinical team is of utmost importance [35]. This collaboration enables optimal interpretation of test results and ensures that they are integrated into the patient’s treatment plan effectively. Ongoing education for both clinicians and laboratory personnel is vital to reinforce the appropriate use of microbiological testing and to enhance understanding of how test results should inform clinical decisions.

Highly sensitive laboratory results, which sometimes detect asymptomatic carriers, can often lead clinicians to prescribe unnecessary antibiotics, contributing to problems and related costs. Notably, none of the clinicians in our study prescribed penicillin, a highly targeted, narrow-spectrum antibiotic for GAS as recommended [12,13,14]. While rapid diagnostic tests provide faster results, which may influence prescribing decisions, it is also important to recognize that relying solely on cultures without rapid testing may sometimes negate the need for antibiotic therapy. For instance, patients may show clinical improvement within 24 h post-consultation, by the time culture results become available, negating the need for antibiotic therapy. This underscores the need for a balanced approach in utilizing diagnostic tests, ensuring they are used appropriately in the clinical context to improve patient outcomes while minimizing unnecessary interventions.

Ultimately, diagnostic stewardship is critical in ensuring that testing is performed only when clinically indicated, aligning with national guidelines. This approach not only optimizes patient care but also contributes to more effective use of healthcare resources, supporting broader public health goals in combating antibiotic resistance.

## Figures and Tables

**Table 1:** Comparison of the results of the culture with RADT and NAAT.

		RADT Positive for Gas?		NAAT Positive for Gas?	
		**No**	**Yes**	**No**	**Yes**
Reference method: Culture Positive for Gas?	No	56	0	54	2
	Yes	5	21	0	26

Table 1 compares the performance of the RADT and NAAT against throat culture. Abbreviations: GAS = Group A *Streptococcus*.

**Table 2:** RADT results compared with the abundance of the colonies on the culture mediums.

Testing Method	Result	Semi-Quantification of Colonies on Culture Media				
		**<10 Colonies**	**1/4 (+)**	**2/4 (++)**	**3/4 (+++)**	**4/4 (++++)**
RADT	NEG	3	0	1	1	0
	POS	0	1	2	14	4
Total Positive TC		3	1	3	15	4

Table 2 correlates RADT results with the semi-quantification of culture mediums. Abbreviations: TC = Throat Culture; NEG = Negative; POS = Positive.

**Table 3:** Total profit calculated based on the total cost and total reimbursement. All prices are in Euros (value-added tax included).

Items		Culture Alone	RADT + Culture	NAAT Alone	NAAT + Culture
Labor cost ¹		9.53	15.59	1.73	11.26
Eswab cost		Identical	Identical	Identical	Identical
Culture medium		0.38	0.38	NA	0.38
Analysis/test		NA	2	22	22
MALDI-TOF ID		0.2	0.2	NA	0.2
Total cost:		10.11	18.17	23.73	33.84
INAMI Reimbursement	CULTURE	B250 = 2.16	B250 = 2.16	NA	B250 = 2.16
	RADT/NAAT	NA	B250 = 2.16	B250 = 2.16	NA
Flat rate ²		21.2	21.2	21.2	21.2
Total INAMI reimbursement ²		23.36	25.52	23.36	23.36
Total difference		13.93	7.35	−0.37	−10.48

Table 3 presents all parameters taken into account in the cost calculation of each testing methodology and reimbursement by the Belgian National Institute for Health and Disability Insurance (INAMI). Abbreviations: INAMI = National Institute for Health and Disability Insurance; B = The B value represents the base unit used for calculating laboratory test reimbursements according to INAMI rates in Belgium, where one B unit represents 0.03456 Euros; NA = Not applicable. ¹ Calculated by considering 2023 labor cost (one hour = 52 Euro TVA included). ² Calculated in the scenario in which there is no other analysis.

**Table 4:** Correlation between McIsaac score, throat culture (TC) results, and practitioners’ management.

McIsaac Score [18]	n TC		Action Sggested by McIsaac et al. Based on the Score [18]	n Action Taken by Practitioners			Total
	**NEG**	**POS**		**No ET**	**ET**	**TIPR**	
−1,0,1	10	1	No further testing or antibiotics	8	0	3	11
2	19	6	Optional RADT/culture and treat if positive	12	7	6	25
3	9	8	Consider RADT/culture and treat if positive	4	1	12	17
4,5	15	14	Consider RADT/culture; empiric antibiotics if clinically needed.	7	11	11	29
Total	54	28		31 (12.90% TC+)	19 (42.10% TC+)	32 (50.0% TC+)	82

Table 4 presents the correlation between McIsaac score, throat culture (TC) results, and practitioners’ management. Abbreviations: TC = Throat culture; n = number; NEG = Negative; POS = Positive; ET = Empirical treatment; TIP = Treatment if positive result; % TC+ = percentage of positive throat culture among them.

## Data Availability

Data supporting this study can be obtained upon request from the authors.

## Abbreviations

The following abbreviations are used in this manuscript:

GAS	Group A *streptococcus*
RADT	Rapid antigen detection test
NAAT	Nucleic acid amplification test
TC	Throat culture
